# The Risk of Trigeminal Neuralgia Following Osteoporosis

**DOI:** 10.3390/medicina58030447

**Published:** 2022-03-18

**Authors:** Yu-Feng Su, Chieh-Hsin Wu, Wei-Ting Wang, Ann-Shung Lieu

**Affiliations:** 1 Department of Surgery, Division of Neurosurgery, Kaohsiung Medical University Hospital, Kaohsiung 807, Taiwan; suyfeng2000@gmail.com (Y.-F.S.); wujoeys@gmail.com (C.-H.W.); 2 Department of Surgery, School of Medicine, College of Medicine, Kaohsiung Medical University, Kaohsiung 807, Taiwan; 3 National Defense Medical Center, Department of Radiology, Tri-Service General Hospital, Taipei City 11490, Taiwan; a8915g@gmail.com

**Keywords:** cohort study, osteoporosis, population, trigeminal neuralgia (TN)

## Abstract

*Background and objectives*: Managing people with trigeminal neuralgia (TN) and osteoporosis is challenging due to their debilitating conditions. Currently, the exact association between TN and osteoporosis in patients remains unknown, although there is potential overlapping of pathophysiological mechanisms. In response, we calculated TN risk in patients who have osteoporosis. *Materials and Methods*: 45,393 patients aged over 50 years diagnosed with osteoporosis were matched with 45,393 non-osteoporosis patients aged over 50 years (1:1 ratio) who were used as the control group, using data from 1996 to 2010 from Taiwan’s National Health Insurance Research Database. The cumulative incidences of subsequent TN and the hazard ratio were estimated using Cox proportional hazards modeling and the Kaplan–Meier method, respectively. *Results*: Among the total sample, 333 patients were diagnosed with TN during the follow-up period: 205 in the osteoporosis cohort and 128 in the control cohort. Through covariate adjustment, the overall TN incidence showed a 1.80-fold increase in the osteoporosis cohort in comparison with the control cohort (0.60 vs. 0.18 per 1000 person-years, respectively). The High Charlson Comorbidity Index, hypertension, and migraines were risk factors of TN. *Conclusions*: Osteoporosis patients had a higher TN risk than that of the control cohort. Therefore, early recognition of pain and symptoms in osteoporotic people may help to identify possible TN patients who need prompt therapy.

## 1. Introduction

Trigeminal neuralgia (TN) was first described in the first century AD by Aretaeus and is well recognized as one of the most common cranial nerve diseases [1]. TN is a pain disorder with recurrent attacks of unilateral shooting or electric shock-like pain followed by pain-free periods. Precipitated by innocuous stimuli to the face, the pain occurs along one or more divisions of the trigeminal nerve [2]. The condition can impair the activities of daily living and give rise to depressive disorders or other comorbidities. The annual incidence of TN was reported as being 4.3 per 100,000 people in Minnesota between 1945 and 1984, and as high as 27 per 100,000 people in the United Kingdom between 1992 and 2002 [3,4,5]. The disease is reportedly more prevalent in females; the male-to-female ratio is 1:1.73. The pain attack often arises in patients older than 50 years of age, with peak incidence occurring between 50 and 70 years [6]. In the diagnosis of TN, which is usually dependent on the patient’s medical history, it is classified as either classic TN or symptomatic trigeminal neuropathy. The majority of TN cases are classified as classic TN, in which the cause is unidentifiable. Classic TN is characterized by microvascular compression of the root entry zone of the trigeminal nerves at the brain stem. A minority of TN cases are classified as symptomatic trigeminal neuropathy, in which the cause is an underlying lesion, such as trauma, a brain tumor, jawbone cavitation [7,8], or multiple sclerosis [2].

Osteoporosis is defined as a systemic skeletal disease specified with a combination of poor quality of bone and/or low bone mineral density (BMD) by the National Institutes of Health conference statement. As a result, bone strength is diminished, and fracture risk is increased [9]. Osteoporosis is very prevalent in the entire population of individuals in the same age range affected by TN [3,10,11,12]. Approximately 50% of women and 20% of men among people aged 50 and older tend to have fractures at some point in their lives [13]. Both TN and osteoporosis are debilitating conditions that can impose a large socioeconomic burden and can impact life quality [10,11]. 

Both osteoporosis and TN share similar pathophysiological mechanisms and risk factors. Firstly, both their risks are positively associated with aging, have a similar age of onset [3,10,11], and, both occur more frequently in females than in males. Secondly, because TN patients often have vascular diseases such as atherosclerosis and arterial hypertension, a vascular theory for TN etiology has emerged [3,4,12]. Osteoporosis and atherosclerosis share some common features, including oxidized lipid accumulation and endothelial dysfunction [14]. Studies suggest that patients with hypertension have a far greater risk for both TN and osteoporosis [4,6]. In addition, chronic inflammation involving pro-inflammatory cytokines is known to play a major role in the development of both long-term conditions [14]. Wu et al. (2017) previously suggested that osteoporosis easily develops in patients with the chronic inflammatory skin disease atopic dermatitis [15]. The release of IL-1, IL-6, NO, and TNF-α, and the interaction of RANKL/RANK can regulate the balance between osteoblasts and osteoclasts [16]. Additionally, vitamin D not only maintains bone mineralization [17] but also modulates pain intensity in TN patients [18]. Finally, loss of jawbone may be a presentation of osteoporosis [19,20], and jawbone cavitation is also considered as a possible pathogenesis of TN [7,8,21]. Therefore, we assumed that osteoporosis is correlated with TN and performed a population-based retrospective cohort study. We used Taiwan’s National Health Insurance Research Database (NHIRD) to determine the connection between osteoporosis and subsequent TN risk.

## 2. Materials and Methods

### 2.1. Database

The compulsory single-payer health insurance system implemented by the Taiwan Bureau of National Health Insurance (BNHI) in 1995 currently provides health coverage to approximately 99% of the 23.74 million Taiwan residents. The National Health Research Institute is in charge of the administration of the NHIRD, an encoded secondary database used by researchers to conduct medical studies. The NHIRD contains health claims and administrative data collected by the BNHI, including medical records of ambulatory and inpatient care, with information of drugs dispensed to patients through contract pharmacy services. As patient identification numbers are scrambled for all data contained in the NHIRD, researchers are given access to relevant claims data, such as gender, demographic status of insured people, date of birth, medication prescriptions, and registry of medical services. This study analyzed LHID2010, a subset of claims data of the NHIRD, consisting of records of 1,000,000 randomly selected beneficiaries who had received medical treatment between1996 and 2010. This study classified all diseases by using the diagnostic codes of the International Classification of Diseases, Ninth Revision, Clinical Modification (ICD-9-CM).

### 2.2. Study Population

The cohort study obtained data of 45,393 patients aged 50 years and over with a diagnosis of osteoporosis (ICD-9-CM code 733.0) from 1996 to 2010. To ensure data precision, the enrollment criteria were as follows: osteoporosis diagnosed in at least 1 inpatient or 2 ambulatory services, at least one BMD examination, and an ICD-9 code confirmed by an orthopedic surgeon. The index date was the date of the initial osteoporosis evaluation. TN (ICD-9-CM code 350.1) in this study was defined as TN diagnosed in at least 1 inpatient or 2 ambulatory services, and neurologists assigned the ICD-9 code. In each case, the diagnosis date was the date when the osteoporosis was first diagnosed. The control group was set a “pseudo date of diagnosis”, meaning the date of diagnosis in the matched subjects.

Excluding criteria designed to detect only patients with classic TN were as follows: TN diagnosis on or before the index date; age <50 years; incomplete information; and any previous diagnosis of stroke (ICD-9-CM codes 430–438), benign or malignant neoplasm of the nervous system (ICD-9-CM codes 191, 192 and 225) [22], or multiple sclerosis (ICD-9-CM code 340).

**Figure 1:**
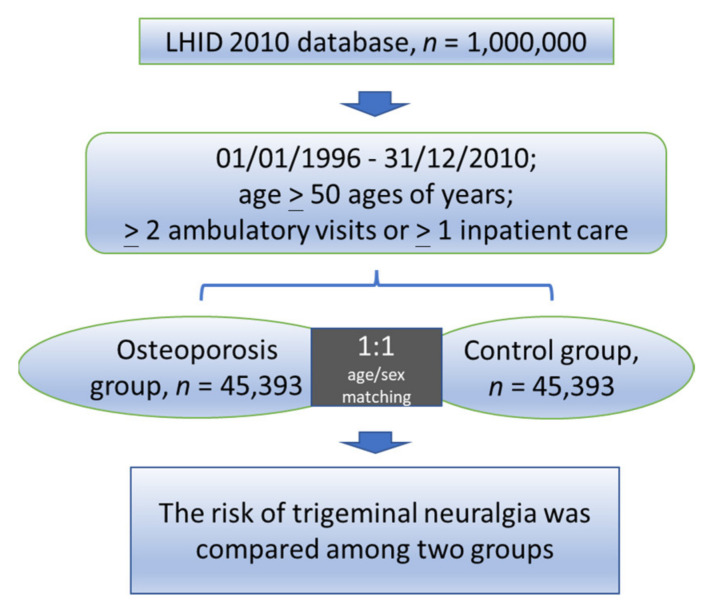
Flow diagram of the study. LHID = Longitudinal Health Insurance Database.

The 1:1 ratio of osteoporosis to non-osteoporosis patients was maintained to enhance the statistical power and to ensure a sufficient number of TN subjects for stratified analyses. The non-osteoporosis cohort of patients was chosen through simple random sampling in which insured people without osteoporosis were chosen randomly from LHID 2010 and matched using a propensity score method with an osteoporosis patient in the same categories of gender and age. Therefore, 45,393 people were selected for the non-osteoporosis cohort. Figure 1 shows a flow chart of the study steps.

### 2.3. Outcome and Comorbidities

All patients in both the osteoporosis and non-osteoporosis groups were followed up until either TN was diagnosed, they applied for insurance withdrawal, or until the end of 2010. Baseline comorbidities were clarified by ICD-9-CM codes, which included chronic periodontitis (ICD-9-CM code 523.4); hypertension (ICD-9-CM codes 401–405); chronic liver disease (ICD-9-CM codes 571.2, 571.4-6, 456.0-21, and 572.2-8); hyperlipidemia (ICD-9-CM code 272); diabetes mellitus (ICD-9-CM code 250); chronic kidney disease (ICD-9-CM codes 582, 583, 585, 586, and 588); migraine (ICD-9-CM code 346); chronic pulmonary disease (ICD-9-CM codes 490–496); alcohol-attributed disease (ICD-9-CM codes 291.0-9, 303, 305.0, 357.5, 425.5, 535.3, 571.0-3, 980.0, and V11.3); coronary artery syndrome (ICD-9-CM codes 410–414); and fractures, including hip fractures (ICD-9-CM code 820), vertebral fractures (ICD-9-CM codes 805.2–805.9), wrist fractures (ICD-9-CM code 813), and humeral fracture (ICD-9-CM code 812). A score from the Charlson Comorbidity Index (CCI) was utilized to elevate the comorbidities severity, i.e., peripheral vascular disease, congestive heart failure, myocardial infarction, any malignancy, metastatic solid tumor, dementia, diabetes, cerebrovascular disease, rheumatic disease, renal disease, chronic pulmonary disease, peptic ulcer disease, liver disease, hemiplegia or paraplegia, acquired immune deficiency syndrome, and human immunodeficiency virus infection. Furthermore, the scores were classified into four levels: 0, 1–2, 3–4 and ≥5.

### 2.4. Statistical Analyses

We used a chi-square test to compare clinical characteristics and distributions of categorical demographics between the cohort of osteoporosis patients and the control cohort. The Kaplan–Meier method was used to estimate cumulative incidence, and the differences between the curves were tested using a 2-tailed log-rank test. Univariable and multivariable Cox proportional hazard regression models were used to investigate the hazard ratio (HR), and a 95% confidence interval (CI) for TN if the proportional hazards assumption was satisfied. The Wilcoxon rank-sum test and Student *t*-test were appropriately used to compare follow-up time and mean age among the two cohorts. For osteoporotic patients, survival was examined until either the end of 2010, hospitalization, or an ambulatory visit for TN, whichever came first. TN incidence rates were estimated in 1000 person-years and compared between the two groups. We adjusted the multivariable Cox models for gender, fractures, age, score of CCI, and the relevant comorbidities (coronary artery syndrome, hyperlipidemia, migraine, chronic periodontitis, chronic kidney disease, chronic pulmonary disease, hypertension, diabetes mellitus, chronic liver disease, and alcohol-attributed disease). Statistical significance was considered as a *p*-value (2-tailed) of <0.05. The SAS statistical package (version 9.4; SAS Institute Inc., Cary, NC, USA) was used to perform data analyses.

## 3. Results

### 3.1. Baseline Characteristics of Subjects with and without Osteoporosis

The baseline comorbidities and demographic characteristics in the two groups are shown in Table 1. In the osteoporosis cohort, female patients were the majority (79.93%). In the osteoporosis group, the percentage of patients with the morbidities mentioned below was significantly higher than that in the non-osteoporosis cohort. In addition, higher CCI scores were calculated in the osteoporosis cohort.

During a median 3.8-year observation time, 205 (0.45%) patients in the osteoporosis cohort had TN (interquartile range = 1.8–7.0). Of the 45,393 gender- and age-matched patients in the non-osteoporosis cohort, only 128 (0.28%) had TN during a median 7.5-year observation time. During the 15-year follow-up, the TN incidence significantly increased in the osteoporosis cohort (*p* < 0.001). In subsequent years, the development period of TN was significantly shorter in the osteoporosis cohort (3.8 years) than in the control cohort (7.5 years).

### 3.2. Incidence and Risk of TN

The incidence of TN and risks analyzed using comorbidity, age, and gender are stratified in Table 2. During the follow-up period, after adjusting for gender, age, fractures, CCI, and related comorbidities, the osteoporosis group had a 1.80 times higher overall TN risk than that of the non-osteoporosis cohort (0.60 vs. 0.18 per 1000 person-years, respectively).

The gender-specific analyses revealed that, in both groups, TN incidence was higher in female patients than in male patients (0.61 vs. 0.57 per 1000 person-years, respectively, in the osteoporosis group, and 0.2 vs. 0.18 per 1000 person-years, respectively, in the non-osteoporosis group). In both genders, risk of a TN attack was significantly higher in the osteoporosis cohort than in the non-osteoporosis cohort (adjusted HR = 1.83, 95% CI: 1.38–2.42 for female; adjusted HR = 1.90, 95% CI: 1.03–3.53 for male). Moreover, the TN incidence was consistently increased in the osteoporosis cohort in all age subgroups, and the incidence rate elevated with aging. Even so, the risk of a TN attack decreased with aging, and the age-stratified risk analysis revealed that higher TN risk was found in osteoporosis patients between 50 and 60 years (adjusted HR = 1.87, 95 % CI = 1.23–2.85, *p* < 0.05) than in patients aged 70 years and older (adjusted HR = 1.78, 95 % CI = 1.21–2.63, *p* < 0.05). In spite of comorbidity, higher TN risk was calculated in osteoporosis patients than in control patients. Besides the presence of comorbidity, the risk of TN contributed by osteoporosis decreased.

**Figure 2:**
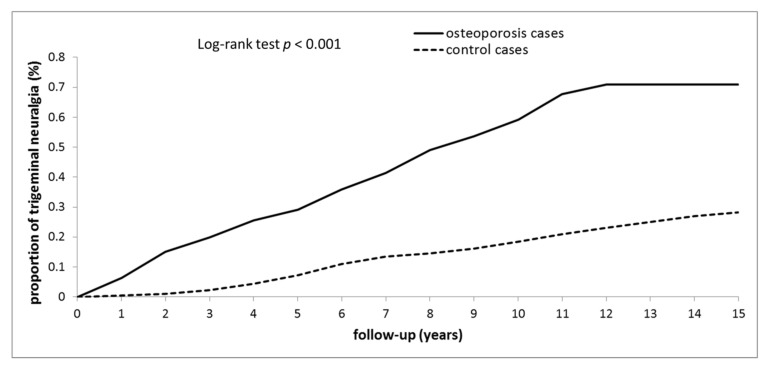
Cumulative incidence of trigeminal neuralgia among people with osteoporosis and controls without osteoporosis.

In Figure 2, the Kaplan–Meier plots for the TN cumulative incidence are compared between the osteoporosis and control cases at the 15 years follow-up. The figure reveals that the TN cumulative incidence was significantly higher in the osteoporosis group than in the control group (log-rank test *p* < 0.001).

### 3.3. Risk Factors for TN in Osteoporosis Cohort

Table 3 reveals the results of Cox regression analysis, which indicates that the TN risk factors in the osteoporosis patients were migraine (adjusted HR = 4.91, 95% CI = 3.62–6.67), hypertension (adjusted HR = 1.55, 95% CI = 1.03–2.34), and high CCI (adjusted HR = 1.28, 95% CI = 1.05–1.56).

## 4. Discussion

In this research, we reported a correlation between TN and osteoporosis, which continued even after adjustment for comorbidities. To the best of our knowledge, this is the first research to conduct a population-based study of the association between osteoporosis and subsequent TN worldwide. In this analysis, TN developed in 205 (0.45%) osteoporosis patients and in 128 (0.28%) patients without osteoporosis during the follow-up. After adjusting for possible confounding factors, TN risk showed a 1.80-fold increase in the cohort with osteoporosis compared with the control cohort. TN risk was particularly high in osteoporosis patients with migraine, hypertension, or high CCI.

In our study, the impact of osteoporosis on the risk of TN increased differently in the two genders and was more prominent in the patients aged between 50–60 years. In addition, women and the elderly had a relative higher incidence of TN, which is consistent with a previous article [23]. Regardless of whether comorbidity co-existed or not, osteoporotic patients had a higher TN risk than non-osteoporosis patients. However, the risk of TN contributed by osteoporosis decreased with the concomitant comorbidity. Since several comorbidities may contribute to TN, the impact of osteoporosis on TN may not be significant with the presence of other comorbidities. Moreover, the incidence of TN increased with concomitant migraine, hypertension, and high CCI. Based on the event rate, this study has more than 99% power (α = 0.05) to detect a significant association between osteoporosis and the risk of TN. These results indicated that the existence of osteoporosis implied possible concomitant TN, suggesting a primary role of osteoporosis in the etiology of TN.

Although the exact underlying mechanisms of the correlation between TN and osteoporosis are rather difficult to explore, the following interpretations are possible. Firstly, migraine is regarded to be a strong risk factor for developing TN. In a recent study by Lin et al., a comparison between 137,529 migraine patients enrolled during 2005–2009 and controls matched 1:1 by age and gender found that the migraine group had a higher incidence of TN. The HR for TN was 6.72 (95% CI, 5.37–8.41; *p* < 0.001). The authors hypothesized that when a migraine attack occurred, the inflammatory neuropeptides were released, and trigeminal vascular system excitation caused the nearby trigeminal neuropathy to cause further demyelination [5]. Moreover, our previous study showed that osteoporosis increased the risk of migraine 1.37-fold when osteoporosis patients were compared to non-osteoporosis patients after adjusting for possible confounding factors. We assumed that inflammation and magnesium both played critical roles in the interplay between osteoporosis and migraine. Combining these results, it can be supposed that people with osteoporosis tend to have trigeminal neuralgia [24]. Secondly, the correlation of blood pressure regulation and calcium metabolism is a major link between hypertension and BMD. Some reports showed that hypertension is correlated with abnormal calcium metabolism, which results in the leakage of bone calcium [25,26,27] that is eventually excreted by the kidneys in the form of urine [28]. Other research papers showed an association between hypertension and subsequent hypercalciuria, and an association between hypercalciuria and further osteoporosis [29,30]. Hypertension is a risk factor for osteoporosis and a suspected risk factor for TN [4,6]. In an analysis of data included in the Taiwan NHIRD for 138,492 hypertension patients, S.-L. Pan et al. reported that hypertension patients have an elevated TN risk (HR: 1.51, 95% CI: 1.19–1.90). The authors hypothesized that by exacerbating arterial tortuosity at the brain stem, hypertension increased the risk of neurovascular compression, which then increased the risk of TN [4]. Since osteoporosis and TN share similar pathophysiological mechanisms and risk factors, the investigated association between osteoporosis and TN risk in this paper is unsurprising. Thirdly, calcitonin gene-related peptide (CGRP) may play a critical role. This neuropeptide is distributed widely in the peripheral and central nervous systems and is considered the most important neuropeptide in the trigeminal sensory pathways [31]. In Qin et al., CGRP expression was analyzed in the peripheral blood and cerebrospinal fluid of 20 primary TN patients. The expression of CGRP in those samples from the primary TN patients significantly differed from that in the control patients [32]. In addition to this, increased CGRP expression during a TN attack may also decrease the pain threshold in local regions [33]. Another study showed that CGRP injected into evirated rats apparently modifies the release of osteoblastic cytokines and indirectly regulates suppression of bone resorption by osteoclasts [34]. In another study of 75 female patients aged 34–70 years complaining of back pain, Lin et al. revealed that the osteoporosis patients had a higher plasma level of CGRP [35]. Finally, the higher CGRP level in the osteoporosis group apparently increased the incidence of TN by decreasing the threshold for TN. Therefore, we hypothesized that osteoporosis may increase TN risk by increasing CGRP expression.

The strongest element of our study is the assessment of a population-based dataset with large sample sizes to investigate an interplay between osteoporosis and TN risk. Even so, this study has some limitations. Firstly, both TN and osteoporosis were defined according to ICD-9-CM codes from claim records. Therefore, one of the study limitations is the uncertainty of accurate disease coding in the dataset, which mainly depends on the individual physician’s performance. Nevertheless, the BNHI organizes regular document scrutinization of medical specialists to reduce error rates for medical coding in Taiwan. Furthermore, the NHIRD has previously been used for various scientific studies in recent years [36]. A smoking habit influences the processing of TN pain at the supraspinal/brain stem level [37]. Another limitation is that the NHIRD lacked detailed data for possible TN trigger factors, such as smoking, dietary habits, caffeine intake, exercise capacity, alcohol consumption, and body mass index, which may also compromise our present results. Thirdly, most inhabitants of Taiwan are ethnic Chinese; further exploration is necessary to determine whether our results can be generalized to other ethnicities. Finally, statistical significance does not necessarily mean clinical significance, and further clinical studies are needed to confirm this relationship.

## 5. Conclusions

In summary, by using a population-based cohort design, this study demonstrated that osteoporotic patients have a higher risk of TN. Medical doctors should keep in mind that osteoporosis is a possible TN risk factor. In patients with osteoporosis, early identification of pain helps and may prevent future TN progression. Additional work is needed to identify the underlying mechanisms of this relationship.

## Figures and Tables

**Table 1:** Baseline characteristics between people with and without osteoporosis.

Variables	Osteoporosis		*p*-Value
	Yes (*n* = 45,393)	No (*n* = 45,393)	
Trigeminal neuralgia patients, *n* (%)	205 (0.45)	128 (0.28)	<0.001
Period of developing trigeminal neuralgia median (IQR), years	3.8 (1.8–7.0)	7.5 (4.9–11.0)	<0.001
Mean age at diagnosis of trigeminal neuralgia, years	64.3 (8.9)	67.2 (10.3)	<0.001
Age group, *n* (%)			
50–60	16,782 (36.97)	16,782 (36.97)	
60–70	14,598 (32.16)	14,598 (32.16)	
>70	14,013 (30.87)	14,013 (30.87)	1.000
Gender, *n* (%)			
Men	9112 (20.07)	9112 (20.07)	
Women	36,281 (79.93)	36,281 (79.93)	1.000
Charlson Comorbidity Index, *n* (%)			
0	1801 (3.97)	8160 (17.98)	
1–2	9943 (21.90)	16,099 (35.47)	
3–4	13,169 (29.01)	11,273 (24.83)	
≥5	20,480 (45.12)	9861 (21.72)	<0.001
Comorbidity, *n* (%)			
Hypertension	34,327 (75.62)	25,583 (56.36)	<0.001
Diabetes mellitus	18,670 (41.13)	13,099 (28.86)	<0.001
Hyperlipidemia	28,693 (69.21)	21,053 (46.38)	<0.001
Migraine	3011 (6.63)	1358 (2.99)	<0.001
Chronic periodontitis	18,656 (41.10)	16,225 (35.74)	<0.001
Chronic kidney disease	10,471 (23.07)	5592 (12.32)	<0.001
Chronic pulmonary disease	21,199 (46.70)	12,386 (27.29)	<0.001
Chronic liver disease	19,053 (41.97)	12,934 (28.49)	<0.001
Coronary artery syndrome	6700 (14.76)	3206 (7.06)	<0.001
Alcohol-attributed disease	1163 (2.56)	993 (2.19)	<0.001
Humeral fracture	2343 (5.16)	1094 (2.41)	<0.001
Wrist fracture	4097 (9.03)	1624 (3.58)	<0.001
Vertebral fracture	10,212 (22.5)	1355 (2.99)	<0.001
Hip fracture	3580 (7.89)	676 (1.49)	<0.001

IQR: interquartile range; SD: standard deviation.

**Table 2:** Trigeminal neuralgia risk and analyses using demographic characteristics and comorbidity among people with or without osteoporosis.

Variables	People with Osteoporosis		People without Osteoporosis		Compared to Non-Osteoporosis Group	
	Trigeminal Neuralgia	Rate	Trigeminal Neuralgia	Rate	Crude HR (95% CI)	Adjusted HR * (95% CI)
Overall	205	0.60	128	0.18	3.12 (2.46–3.96) ^#^	1.80 (1.38–2.34) ^#^
Gender						
Men	32	0.57	25	0.18	3.27 (1.84–5.83) ^#^	1.90 (1.03–3.53) ^#^
Women	173	0.61	103	0.20	3.11 (2.39–4.04) ^#^	1.83 (1.38–2.42) ^#^
Stratify by age						
50–60	77	0.56	36	0.14	3.81 (2.54–5.72) ^#^	1.87 (1.23–2.85) ^#^
60–70	66	0.57	38	0.17	3.25 (2.16–4.89) ^#^	1.84 (1.21–2.79) ^#^
>70	62	0.70	54	0.26	2.67 (1.83–3.89) ^#^	1.78 (1.21–2.63) ^#^
Comorbidity ^&^						
No	8	0.23	11	0.05	4.20 (1.68–10.49) ^#^	3.09 (1.23–7.75) ^#^
Yes	197	0.64	117	0.23	2.56 (2.01–3.28) ^#^	1.76 (1.35–2.28) ^#^

Abbreviations: 95% CI, 95% confidence interval; HR, hazard ratio. Rate, per 1000 person-years; * calculated by multivariate Cox proportional hazard regression model; ^&^ patients with any examined comorbidities were classified as the comorbidity group; ^#^ *p* < 0.05.

**Table 3:** Significant predictors of trigeminal neuralgia after osteoporosis.

Variables	Adjusted HR * (95% CI)
Migraine	4.91 (3.62–6.67) ^#^
Hypertension	1.55 (1.03–2.34) ^#^
Charlson Comorbidity Index	1.28 (1.05–1.56) ^#^

95% CI, 95% confidence interval; HR, hazard ratio. * Calculated by stepwise Cox proportional hazards regression method. ^#^ *p* < 0.05.

## Data Availability

All data generated or analyzed during this study are included in this published article.
